# Serum Activins and Follistatin during the Treatment of Chronic Hepatitis C Genotypes 1 and 4 and Their Correlations with Viral Load and Liver Enzymes: A Preliminary Report

**DOI:** 10.1155/2014/628683

**Published:** 2014-04-01

**Authors:** Bassem Refaat, Adel Galal El-Shemi, Ahmed Mohamed Ashshi, Adnan AlZanbagi

**Affiliations:** ^1^Laboratory Medicine Department, Faculty of Applied Medical Sciences, Umm Al-Qura University, P.O. Box 7607, Al Abdeyah, Makkah, Saudi Arabia; ^2^Department of Pharmacology, Faculty of Medicine, Assiut University, Assiut 6515, Egypt; ^3^Gastroenterology Department, King Abdullah Medical City, Makkah 21955, Saudi Arabia

## Abstract

*Aims*. To measure the effect of pegylated interferon-**α** therapy on serum activin-A, activin-B, and follistatin and their correlation with viral load and liver fibrosis in chronic hepatitis C (CHC). *Methods*. This study was cross-sectional and sera were collected from 165 participants classified into 7 groups: 40 healthy negative control, 33 treatment naïve patients as positive control, 19 patients at week 4, 22 at week 12, and 19 at week 24 of treatment initiation and 21 responders and 11 nonresponders at the end of 48-week treatment protocol. Serum candidate proteins were measured using ELISA and liver fibrosis was assessed by AST platelet ratio index (APRI). *Results*. CHC significantly increased activins and decreased follistatin compared to negative control (*P* < 0.05). Activin-A and follistatin levels returned to the levels of negative control group at weeks 4, 12, and 24 following treatment initiation and were significantly different from positive control (*P* < 0.05). Both proteins were significantly different between responders and nonresponders. Activin-A correlated positively and significantly with the viral load and APRI. *Conclusion*. CHC modulates serum activin-A and follistatin and they appear to be influenced by pegylated interferon-**α** therapy. Further studies are needed to explore the role of activins in CHC.

## 1. Introduction

Infection with hepatitis C virus (HCV) is a global health problem and it infects at least 170 million people worldwide with an estimated prevalence of 3 to 4 million newly acquired infections per year [1]. Although future treatment for chronic hepatitis C (CHC) is interferon free and it achieves higher sustained viral response, HCV infection is still currently treated with a weekly injection of pegylated interferon-*α*- (PEG-INF-*α*-) 2a or -2b plus a daily weight-based dose of ribavirin and the recommended duration of treatment for genotypes 1 and 4 is 48 weeks [2, 3]. Furthermore, the cost of the new treatment is estimated to be 60,000–100,000 USD and therefore PEG-INF-*α* may still have a role especially in those patients living in developing countries and for whom access to the new drugs is not definite [4–8].

IFN-*α* alters the immune response in patients with CHC from T helper- (Th-) 2 to a Th1 mediated pattern to favour the eradication of the virus [9–12] and this is achieved through the modulation of several cytokines, including IFN-*γ*, interleukin (IL)-2, IL-6 and IL-10, tumour necrosis factor- (TNF-) *α*, and transforming growth factor-*β* (TGF-*β*) [13–16].

Activins, which are members of the TGF-*β* family, are homodimers of inhibin *β*-subunits (*β*A and *β*B), and the different dimerization of subunits gives rise to three proteins: activin-A (*β*A-*β*A), activin-B (*β*B-*β*B), and activin-AB (*β*A-*β*B), and their biological actions are tightly regulated by their binding protein, follistatin [17]. Activin-A and follistatin are expressed by the hepatocyte and they are involved in the regulation of liver regeneration. The expression of these proteins is altered in a variety of liver pathologies, including viral hepatitis, liver fibrosis/cirrhosis, and hepatocellular carcinoma [18–22].

Activin-A and follistatin also play an important immunoregulatory role in the pathogenesis of inflammatory and fibrotic human diseases [23]. In this concept, activin-A can act as a pro- or anti-inflammatory agent depending on type of the disease and the cellular and immune contexts and its actions are blocked by follistatin [24, 25]. Moreover, activin-A may be a key regulator of humoral immune response and its secretion is up-regulated in activated Th2 clones [26–28].

Little is known about the effect of PEG-INF-*α* based therapy for the treatment of CHC on serum concentrations of activins and follistatin. Therefore, concentrations of activin-A, activin-B, and follistatin in serum samples collected at different times during the treatment protocol for genotypes 1 and 4 were compared with those obtained from healthy and treatment naïve patients with CHC genotypes 1 and 4. We also investigated whether serum concentrations of the candidate molecules differ between treatment responder and nonresponder and correlation studies were performed between the candidate molecules, viral load, and liver function parameters.

## 2. Patients and Methods

### 2.1. Ethical Approval

The study was approved by the Institutional Review Board and Ethics Committee of King Abdullah Medical City (IRB 12-028). All samples were collected following obtaining informed written consent from all the participants.

### 2.2. Study Design

The trial was a cross-sectional and serum samples were collected from 40 healthy blood donors and they served as negative control (NC) group. The NC group consisted of 20 males and 20 females (age range: 30–60 years). The participants did not report any current acute or chronic medical condition, history of hospitalisation, and no medication for significant clinical disease. Additionally, laboratory results for their haematological, biochemical, and metabolic parameters were within normal range.

Serum samples were also collected according to the inclusion and exclusion criteria (Table 1) from 6 different groups of patients with a total number of 125 patients diagnosed with CHC and for whom polymerase chain reaction following reverse transcription was positive for HCV RNA. The 6 groups were defined according to the time of their sample collection: positive control (PC) group included 33 infected patients who did not start their treatment protocol. The 4 weeks (4 W) group included 19 patients at week 4 after the initiation of 48 weeks of treatment protocol with pegylated-interferon-*α* based therapy who achieved a rapid viral response. The 12 weeks (12 W) group consisted of 22 patients at week 12 after the start of the treatment protocol. The 24 weeks (24 W) group consisted of 19 patients at week 24 following the initiation of treatment. The responder (R) group included samples collected at week 48 from 21 patients who achieved end of treatment viral response and who remained negative after 6 months from the termination of the treatment protocol. The non-responder (NR) group included samples collected from another 11 patients who were positive for HCV by PCR at the end of a 48-week treatment protocol.

All treated patients received PEG-INF-*α*-2a (Pegasys, Roche, Basel, Switzerland) at a dosage of 180 *μ*g per week in combination with daily dose of ribavirin (Copegus, Roche) based on the body weight (1000 mg if <75 kg or 1200 mg if ≥75 kg).

The results of liver function parameters and viral load at the time of sample collection were performed as part of the routine laboratory follow-up.

### 2.3. Calculation of AST/Platelet Ratio Index (APRI)

APRI was calculated using the following equation: (AST/upper limit of normal)/platelet count (×10^9^/L) × 100. The interpretation of the APRI results was performed according to previously published studies [29, 30]. Briefly, index ≤0.5 indicated normal liver with no or minimal fibrosis, >0.5 and ≤1.5 Progressive fibrotic stages (e.g. Metavir F1-to-F4) and >1.5 indicated liver cirrhosis. Only samples with ≤1.2 APRI were included into the study to avoid collecting samples from patients with liver cirrhosis.

### 2.4. Enzyme Linked Immunosorbent Assay (ELISA)

ELISA was used for quantitative measurement of human activin-A (R&D systems, Minneapolis, USA), activin-B (Uscn Life Science Inc., Hubei, China), and follistatin (R&D systems, Minneapolis, USA). All samples were processed in duplicate and according to the manufacturer's instructions. The optical density of the plates was measured within 10 min using a plate reader at 450 nm and correction at 560 nm as recommended by the manufacturer.

As reported by the manufacturer, the lowest detection limit of activin-A by the used kit is 3.7 pg/mL and the upper limit is 1500 pg/mL. The intra-assay and interassay precisions of the kit are 4.3% and 5.8%, respectively. The kit cross reacts by 0.2% and 0.45% with inhibin-A and activin-AB, respectively. The detection range of the activin-B kit is 15.6–1000 pg/mL and the minimum detectable dose is 6.4 pg/mL. The intra-assay and interassay precisions are <10% and <12%, respectively. Cross-reactions with the other activin/inhibin isoforms were not detected by the manufacturer.

### 2.5. Activin(s)/Follistatin Ratio Index

Activin-A/follistatin ratio index (AFRI), activin-B/follistatin ratio index (BFRI), and activins/follistatin ratio index (ASFRI) were calculated as follows, respectively: [activin-A/follistatin × 100], [activin-B/follistatin × 100], and [(activin-A + activin-B)/follistatin × 100].

### 2.6. Statistical Analysis

Statistical analysis of the results was performed using SPSS version 16. Cross-tabulation followed by Chi square (*χ* ^2^) test was used for frequency analysis. According to data normality, either Student's* t*-test or Mann-Whitney* U* test was used to compare between 2 groups. Furthermore, one-way ANOVA followed by LSD post hoc test or Kruskal-Wallis followed by Dunn's post hoc test was used to compare between more than 2 groups depending on the data homogeneity. Correlations were determined using Pearson's test. *P*  value<0.05 was considered significant.

## 3. Results

### 3.1. Demographic Data and Routine Laboratory Parameters

There was no significant difference in the mean age, distribution of gender, viral genotype, viral load at diagnosis, and ALP either between the 7 study groups or within each group according to gender and viral genotype (Table 2). However, serum albumin, AST, ALT, and APRI were significantly different between the study groups (Table 2).

### 3.2. Activins and Follistatin in the Different Study Groups

**Figure 1:**
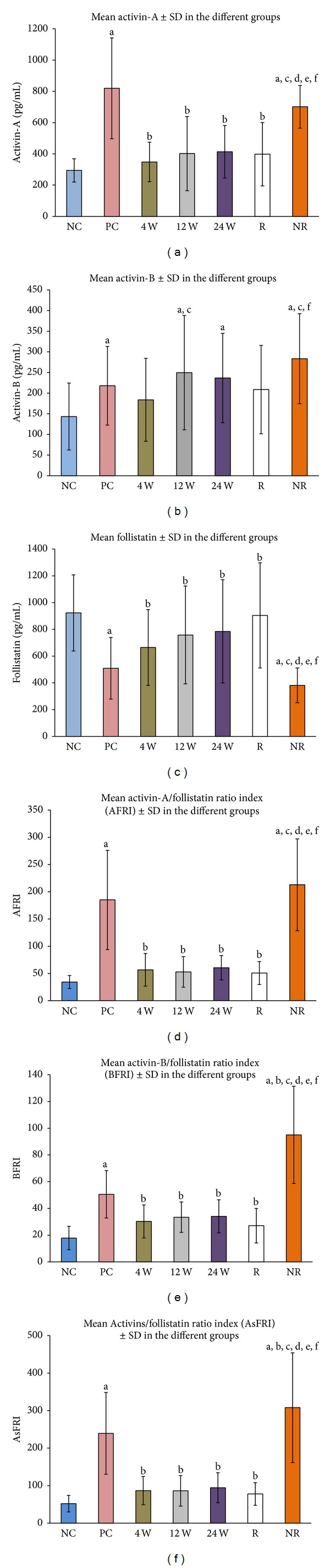
Mean ± SD of serum (a) activin-A, (b) activin-B, (c) follistatin, (d) AFRI, (e) BFRI, and (f) ASFRI in the different study groups (^a^ *P* = 0.05 compared to NC, ^b^ *P* < 0.05 compared to PC, ^c^ *P* < 0.05 compared to 4 W, ^d^ *P* < 0.05 compared to 12 W, ^e^ *P* < 0.05 compared to 24 W, and ^f^ *P* < 0.05 compared to R).

Overall, activin-A, activin-B, follistatin, AFRI, BFRI, and ASFRI showed significant variation between the 7 study groups. CHC with no treatment (PC group) significantly increased serum concentrations of activin-A (*P* = 0.01 × 10^−6^), activin-B (*P* = 0.003), AFRI (0.01 × 10^−6^), BFRI (*P* = 0.02), ASFRI (*P* = 0.0004), and significantly decreased follistatin (*P* = 0.03 × 10^−5^) compared to the NC group (Figure 1).

Following the initiation of treatment, a significant decrease (*P* < 0.05) in the concentrations of activin-A, AFRI, BFRI, and ASFRI was observed at 4 W, 12 W, and 24 W compared to the PC group and the levels were similar to the NC (*P* > 0.05). Significant increase in the concentrations of follistatin (*P* < 0.05) was observed at 12 W and 24 W compared to the PC group and the levels were not different from the NC group (Figure 1). There was no significant change for activin-B following the initiation of treatment when compared to the PC group (*P* > 0.05) (Figure 1). Furthermore, there was no significant change between the 4 W, 12 W, and 24 W in serum levels of activin-A, activin-B, AFRI, BFRI, and ASFRI.

The responder group had serum concentrations of activin-A, AFRI, BFRI, ASFRI, and follistatin at similar levels seen in the NC, 4 W, 12 W, and 24 W groups (*P* > 0.05). However, activin-A (*P* = 0.01 × 10^−4^), AFRI (*P* = 0.001), BFRI (*P* = 0.02), and ASFRI (0.03 × 10^−4^) were significantly decreased and follistatin significantly increased (*P* = 0.01 × 10^−3^) compared to the PC group. There was no significant in activin-B levels when compared to the other groups.

In the nonresponder group, serum activin-A, activin-B, and follistatin were significantly different from all groups except for the PC group, where no significant difference was detected. However, significant differences were detected for AFRI, BFRI, and ASFRI when compared to all groups (Figure 1).

Activin-A and follistatin were significantly higher in male (331.1 ± 65.5 pg/mL and 1111.3 ± 267.8 pg/mL, resp.) compared to female (256.8 ± 70.1 pg/mL and 734.7 ± 185.9 pg/mL, resp.) participants in the control group (*P* < 0.05). However, there was no significant difference (*P* > 0.05) between both genders within each CHC group (data not shown).

### 3.3. Correlation of Serum Activins and Follistatin with Liver Enzymes, Albumin, and Viral Load

**Figure 2:**
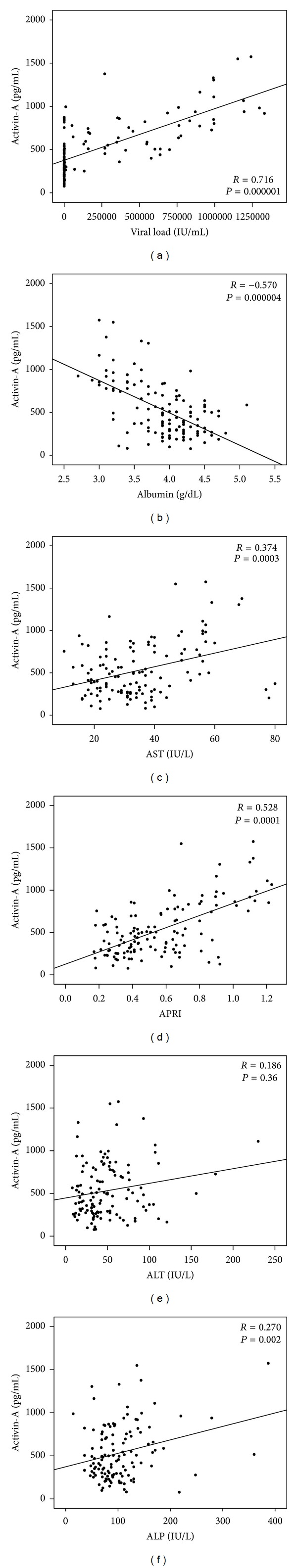
Correlation of serum activin-A with (a) viral load, (b) serum albumin, (c) AST, (d) APRI, (e) ALT, and (f) ALP by Pearson's correlation test.

Activin-A significantly correlated positively with the viral load (*r* = 0.716, *P* = 0.01 × 10^−7^), AST (*r* = 0.374, *P* = 0.0003), and APRI (*r* = 0.528, *P* = 0.01 × 10^−4^) and negatively with albumin (*r* = −0.570, *P* = 0.04 × 10^−5^) (Table 3). Significant positive correlation was also observed between the viral load and AFRI (*r* = 0.604, *P* = 0.01 × 10^−4^) and ASFRI (*r* = 0.534, *P* = 0.03 × 10^−3^) (Figure 2).

## 4. Discussion

This current study is the first to report the effect of PEG-INF-*α* based therapy on serum concentrations of activin-A, activin-B, AFRI, BFRI, ASFRI, and follistatin in patients diagnosed with CHC. This is also the first report to show significant difference in serum concentrations of those proteins between treatment responders and nonresponders in patients infected with HCV genotypes 1 and 4 following PEG-INF-*α* based therapy for 48 weeks. Finally, our results showed a significant correlation for serum activin-A with the viral load and liver function parameters.

Our results suggest that HCV and/or its associated liver fibrosis modulated serum activin-A and follistatin. Additionally, both molecules, but not activin-B, could be modulated through PEG-INF-*α* based therapy in CHC patients.

The expressions of activin subunits and follistatin have been reported in the liver and alteration in their expression has been linked with several liver diseases [18]. Serum activin-A significantly increases in liver fibrosis and cirrhosis induced by viral and non-viral factors [31]. Serum activin-A was linked to viral replication in chronic hepatitis B and hepatitis C [19], and it correlated significantly with liver damage associated with HCV [20]. Hence, it has been suggested that pathological alteration in the hepatic expression of activin and follistatin could lead to impaired liver regeneration [32] and the development of liver fibrosis, cirrhosis, and hepatocellular carcinoma [33]. Our results correlate and support the previous findings as there was a significant decrease in serum follistatin and significant increase in serum activin-A, activin-B, AFRI, BFRI, and ASFRI in patients with CHC and who did not receive treatment compared to healthy controls.

APRI has been proposed as a predictor of liver fibrosis and cirrhosis in CHC to replace liver biopsy in a substantial proportion of patients [34]. Several studies have confirmed the high sensitivity and specificity and significant correlation of APRI with both the stage of liver fibrosis and the grade of activity [29]. The present study showed significant positive correlations of serum activin-A with liver enzymes and APRI and a significant negative correlation with serum albumin, suggesting that the observed increase in activin-A and decrease in follistatin in the “PC” and “nonresponder” groups could be due to the CHC associated liver fibrosis. Therefore, we hypothesize that serum activin-A could be a promising serum marker for the diagnosis of liver fibrosis/cirrhosis associated with CHC. However, further studies are required to support our suggestion.

The current results also showed significant positive correlations for activin-A, AFRI and ASFRI with the viral load, proposing that serum activin-A and follistatin could be modulated by HCV through either the associated liver damage or as part of the host immune response to control the viral infection. Activin-A and follistatin are elements of both innate and humoral immune responses [24, 25] and both proteins have been described in immune response to several pathogens including viruses [35]. Activin-A and follistatin are also important regulators of regulatory T-cells [36], natural killer cells [37], and dendritic cells [38], which are known to play an important role in controlling CHC infection [10]. Furthermore, activin-A has been shown to promote the production of Th2 cytokines, which promote the development of humoral response and the subsequent development of liver fibrosis [9, 10]. Hence, we suggest that the observed changes in in serum activin-A/follistatin could represent the response by the host to the viral damage which triggers an inflammatory response driven by activin-A. However, additional studies are needed to explore whether activin-A and/or follistatin are involved in the immune response to HCV.

PEG-INF-*α* based therapy is a key component of CHC treatment through the induction of a Th1 immune response [39]. INF-*α* promotes Th1 response through the increase in the production of several cytokines by the hepatocyte and immune cells [16]. Additionally, IFN-*α* alters the production of immunoglobulin, decreases T regulatory cell function [40], and stimulates the production of toll like receptors (TLR) [39].

Gathered data from published reports suggest that activin-A/follistatin might be a target for PEG-INF-*α* based therapy for the eradication of CHC. The expressions of TLR-2 and 4 increase significantly following PEG-INF-*α* based therapy in patients with CHC [41] and they are potent regulators of the release of activin-A [42]. In addition, PEG-INF-*α* increases the production of TNF-*α* [43] and decreases serum IL-6 and IL-10 in patients with CHC [14]. These cytokines have also been reported to be regulated by activin-A [36]. Moreover, activin-A has been reported to modulate the release of INF-*γ* [44], which plays an important role in controlling CHC following PEG-INF-*α* based therapy [45]. Consequently, the observed significant alteration in activin-A and follistatin after 4, 12, and 24 weeks of treatment could be mediated by the therapy used. More studies are required to illustrate the kinetics and source of serum activin-A and follistatin during the course of PEG-INF-*α* based therapy.

A limitation for our study is that we adopted a cross-sectional design and performing a longitudinal prospective cohort study would reveal the true kinetics of serum activin-A and follistatin following PEG-INF-*α* based therapy and their correlation with the viral load during the course of treatment. Subsequently, it would allow a better understanding of the effect(s) of the currently used treatment for CHC and viral load on serum activins and follistatin. However, the current study is a preliminary report and we are currently processing future studies with longitudinal design to measure the clinical value of activins and follistatin in the prediction of treatment outcome in CHC.

In conclusion, serum concentrations of activin-A and follistatin are different between patients with and without HCV and at the different stages of IFN therapy. Activin-A and follistatin appear to be influenced by PEG-IFN-*α* based therapy and they seem to correlate with liver damage associated with CHC. Further studies are needed to illustrate the role(s) of activin-follistatin axis in CHC and to explore the effect(s) of PEG-INF-*α* therapy on the expression of activins and follistatin by the hepatocyte.

## Figures and Tables

**Table 1:** Inclusion and exclusion criteria.

Principal inclusion criteria	Principle exclusion criteria
Patient age ≥18 years.	Patient age <18 years.
HCV RNA positive	Previous nonresponders/relapse
No concurrent infection with HBV or HIV	Solid organ transplant (renal, heart, or liver, etc.)
Dual therapy using peg-INF-*α* 2a or 2b with ribavirin	Mono-or triple based therapy
No amendment/modification of treatment protocol	amendment/modification of treatment protocol
Treatment naïve patients	Autoimmune condition (e.g. type 1 DM, rheumatoid arthritis, etc.)
Compensated liver disease (e.g. no liver cirrhosis, failure, or cancer) and APRI ≤1.2	History or current thyroid disease
Acceptable haematological and biochemical indices	Uncontrolled type 2 DM and HTN
No or controlled type 2 diabetes mellitus and hypertension	Concurrent chronic disease (renal failure, coronary heart disease, etc.)

**Table 2:** Demographic and laboratory characteristics of the patients according to study groups.

	NC (*n* = 40)	PC (*n* = 33)	4 W (*n* = 19)	12 W (*n* = 22)	24 W (*n* = 19)	*R* (*n* = 21)	NR (*n* = 11)
Age (years)	39 ± 7.4	49.8 ± 16.3	41.5 ± 13.4	42 ± 11.5	44.9 ± 12.5	46 ± 13.4	42.1 ± 18.1
Gender							
Male	20 (50%)	16 (48.4%)	11 (57.9%)	15 (68.1%)	13 (68.4%)	12 (57.1%)	5 (45.5%)
Female	20 (50%)	17 (51.6%)	8 (42.1%)	7 (31.9%)	6 (31.6%)	9 (42.9%)	6 (55.5%)
Genotype							
G1	ND	14 (42.4%)	10 (52.9%)	10 (47.3%)	10 (55.5%)	11 (52.3%)	3 (27.3%)
G4	ND	19 (57.6%)	9 (41.1%)	12 (52.7%)	9 (45.5%)	10 (47.7%)	8 (72.7%)
Viral load at diagnosis (IU/mL)	ND	1256194 ± 555544	1397057 ± 481355	1089313 ± 617246	999187 ± 400348	1048288 ± 303944	1165716 ± 457946
Albumin (g/dL)	4.4 ± 0.2	3.66 ± 0.46^a^	3.88 ± 0.43^a^	3.84 ± 0.53^a^	4.03 ± 0.23^a^	3.91 ± 0.39^a^	3.95 ± 0.48^a^
ALP (IU/L)	79.4 ± 21.6	120.2 ± 51.8	112.4 ± 35.8	100.6 ± 39.3	85.8 ± 26.5	87.8 ± 32.6	110.09 ± 42.8
ALT (IU/L)	28 ± 11.2	63 ± 25.7^a^	53.3 ± 24.7^a^	56.3 ± 26.2^a^	45 ± 14.3^b^	37.6 ± 20.8^b^	38.9 ± 18.7^b^
AST (IU/L)	21 ± 7.6	43.4 ± 17.2^a^	39.3 ± 16.2	38.3 ± 12.6	31.2 ± 8.3^b,c^	28 ± 10.7^b,c^	42.4 ± 20.4^a,e,f^
APRI	0.37 ± 0.07	0.85 ± 0.29^a^	0.55 ± 0.23^a^	0.77 ± 0.28^a^	0.66 ± 0.2^a,b^	0.65 ± 0.21^a,b^	0.72 ± 0.3^a^

ND = not done, ^a^ *P* < 0.05 compared to NC, ^b^ *P* < 0.05 compared to PC, ^c^ *P* < 0.05 compared to 4 W, ^d^ *P* < 0.05 compared to 12 W, ^e^ *P* < 0.05 compared to 24 W, and ^f^ *P* < 0.05 compared to *R*.

**Table 3:** Results of correlation analysis using Pearson's test for activin-A, activin-B, follistatin, AFRI, BFRI, and ASFRI with viral load, albumin, liver enzymes, and APRI.

	Viral load at sample collection	Albumin	ALP	ALT	AST	APRI
Activin-A						
*R* value	0.716*	−0.570*	0.270*	0.186	0.374*	0.528*
*P* value	0.000000001	0.0000004	0.002	0.36	0.0003	0.000001
Activin-B						
*R* value	0.113	0.107	0.063	−0.146	−0.023	0.157
*P* value	0.21	0.23	0.48	0.1	0.8	0.076
Follistatin						
*R* value	−0.244	−0.065	0.084	−0.072	−0.061	0.051
*P* value	0.007	0.46	0.35	0.42	0.49	0.56
AFRI						
*R* value	0.604*	−0.289	0.098	0.067	0.146	0.233
*P* value	0.000001	0.003	0.27	0.45	0.1	0.008
BFRI						
*R* value	0.222	0.02	−0.052	−0.068	−0.077	−0.069
*P* value	0.01	0.8	0.56	0.44	0.39	0.44
ASFRI						
*R* value	0.534*	−0.219	0.056	0.034	0.100	0.158
*P* value	0.00003	0.01	0.53	0.42	0.26	0.07

*P < 0.01.
